# Implementation of a nurse-led behaviour change intervention to support medication taking in type 2 diabetes: beyond hypothesised active ingredients (SAMS Consultation Study)

**DOI:** 10.1186/1748-5908-9-70

**Published:** 2014-06-05

**Authors:** Wendy Hardeman, Laura Lamming, Ian Kellar, Anna De Simoni, Jonathan Graffy, Sue Boase, Stephen Sutton, Andrew Farmer, Ann Louise Kinmonth

**Affiliations:** 1 Primary Care Unit, Institute of Public Health, Forvie Site, University of Cambridge School of Clinical Medicine, Cambridge Biomedical Campus, Box 113, CB2 0SR Cambridge, UK; 2 Institute of Psychological Sciences, University of Leeds, LS2 9JT Leeds, UK; 3 Department of Primary Care Health Sciences, University of Oxford, OX1 2ET Oxford, UK

**Keywords:** Fidelity, Implementation, Medication adherence, Behaviour change techniques, Process evaluation

## Abstract

**Background:**

Implementation of trial interventions is rarely assessed, despite its effects on findings. We assessed the implementation of a nurse-led intervention to facilitate medication adherence in type 2 diabetes (SAMS) in a trial against standard care in general practice. The intervention increased adherence, but not through the hypothesised psychological mechanism. This study aimed to develop a reliable coding frame for tape-recorded consultations, assessing both *a priori* hypothesised and potential active ingredients observed during implementation, and to describe the delivery and receipt of intervention and standard care components to understand how the intervention might have worked.

**Methods:**

211 patients were randomised to intervention or comparison groups and 194/211 consultations were tape-recorded. Practice nurses delivered standard care to all patients and motivational and action planning (implementation intention) techniques to intervention patients only. The coding frame was developed and piloted iteratively on selected tape recordings until *a priori* reliability thresholds were achieved. All tape-recorded consultations were coded and a random subsample double-coded.

**Results:**

Nurse communication, nurse-patient relationship and patient responses were identified as potential active ingredients over and above the *a priori* hypothesised techniques. The coding frame proved reliable. Intervention and standard care were clearly differentiated. Nurse protocol adherence was good (M (SD) = 3.95 (0.91)) and competence of intervention delivery moderate (M (SD) = 3.15 (1.01)). Nurses frequently reinforced positive beliefs about taking medication (*e.g.*, 65% for advantages) but rarely prompted problem solving of negative beliefs (*e.g.*, 21% for barriers). Patients’ action plans were virtually identical to current routines. Nurses showed significantly less patient-centred communication with the intervention than comparison group.

**Conclusions:**

It is feasible to reliably assess the implementation of behaviour change interventions in clinical practice. The main study results could not be explained by poor delivery of motivational and action planning components, definition of new action plans, improved problem solving or patient-centred communication. Possible mechanisms of increased medication adherence include spending more time discussing it and mental rehearsal of successful performance of current routines, combined with action planning. Delivery of a new behaviour change intervention may lead to less patient-centred communication and possible reduction in overall trial effects.

**Trial registration:**

ISRCTN30522359.

## Background

Trial evaluations of behaviour change interventions in primary care are increasingly common. They have paid considerable attention to processes affecting the validity of the trial design, such as allocation concealment and robust randomisation, and trial parameters, such as inclusion criteria, power and precision of measurement, facilitated by the CONSORT statement [1]. However, trial evaluations rarely include an assessment of the extent to which interventions are delivered and received as planned (fidelity), to what extent they are adapted, and what this means for long-term implementation and impact in routine clinical practice [2,3]. A review of 202 psychosocial treatment evaluations showed that fidelity was only adequately addressed in 3.5% of the treatments [4]; and a review of 162 evaluations of primary and early secondary prevention programs showed that only 24.1% reported fidelity procedures [5]. Fidelity assessment is also rare for behaviour change interventions to support medication adherence. As an example, in a nurse-led medication adherence intervention based on social-cognitive theory among people with HIV/AIDS, Wickersham *et al.*[6] assessed adherence and quality of nurse-patient interaction and concluded that intervention delivery was successful. They reported overall adherence scores rather than scores for intervention components and did not report reliability of the measures.

Fidelity assessment of behaviour change interventions is important for several reasons. When a hypothesised mechanism of effect or logic model has been defined, fidelity can be assessed in relation to hypothesised active ingredients (intervention components assumed to facilitate behaviour change) to demonstrate that they were delivered in the intervention group as planned (treatment integrity) but not in the comparison group (treatment differentiation) [7]. This approach lends itself to tests of theory by showing the extent to which fidelity is linked to outcomes. However, interventions may be effective or ineffective due to factors unrelated to the hypothesised mechanism, for example adaptations to the planned intervention, practitioner characteristics (*e.g.*, engagement), patient characteristics (*e.g.*, literacy levels), relationship between practitioner and patient, and context. An assessment of actual implementation in practice through tape-recording or observation may identify active ingredients and ways in which the intervention worked beyond those hypothesised *a priori*. This could improve practitioner training, fidelity measurement and intervention design, and identify adaptations that may increase faithful implementation and impact in the long term.

Fidelity assessments of behaviour change interventions have focused on delivery, such as proportion of specified components delivered [8], practitioners’ use of communication skills [9] and behaviour change techniques [10,11]. Assessment of participants’ responses during intervention contacts, *e.g.*, engagement, understanding of intervention principles, and their relationship with practitioners is less common [12]. Studies have relied on self report, but providers’ [10] and recipients’ [13] recall are susceptible to bias and they have weak associations with independent assessment such as observation, audio- or video-recording [14]. To our knowledge no guidance exists for how to identify potential active ingredients beyond those specified *a priori* from observing implementation in practice.

This paper reports the use of a novel method to develop a coding frame for the independent, in-depth assessment of the implementation of a behaviour change intervention and standard care to support medication taking in type 2 diabetes. The study was motivated by the principal results of the Support and Advice for Medication Study (SAMS) and analysis of the hypothesised mechanism of effect [15,16]. The trial was designed to improve the documented weak adherence of patients to their oral diabetes medication [17,18], and was based on extensive development work (see [15] for details including justification of theory selection). The trial evaluated an intervention targeting people with type 2 diabetes, delivered by their usual practice nurse. Participants in the comparison group attended a consultation where the nurses enquired about medications and took a blood sample. Intervention patients additionally received an intervention which targeted two hypothesised causes of suboptimal adherence: weak motivation and forgetting [19]. Nurses were trained in the use of behaviour change techniques which were hypothesised *a priori* as active ingredients impacting on a theory-based causal pathway. They included motivational techniques which aimed to strengthen patients’ intention to take medication by targeting underlying beliefs based on the Theory of Planned Behaviour (TPB) [20], and an action planning technique which aimed to translate motivation into action, based on implementation intentions [21]. Intervention training also included patient-centred communication skills. These were not hypothesised as active ingredients but deemed important for building rapport with patients and competent delivery of behaviour change techniques.

An explanatory trial among 211 patients was carried out over 12 weeks after the intervention and comparison consultations, and medication adherence was measured objectively during that period. The intervention group showed a significant increase in the percentage of days when they took the prescribed medication, compared with the comparison group (77.4% of adherent days in the intervention group and 69.0% in the comparison group) [16]. However the intervention did not strengthen self-reported intentions or habits as hypothesised. Thus, either the measures of intention and habit did not capture the mechanism of effect, or the motivational and action-planning techniques improved adherence through a different mechanism, or the nurses did not deliver the intervention as specified. This study was designed to understand how the intervention might have worked, based on a coded analysis of tape-recorded consultations, and additionally using grounded theory to identify any other factors that might have increased medication adherence from listening to tape recordings. Our objectives were (1) to develop a reliable coding frame for tape-recorded consultations, assessing both *a priori* hypothesised and potential active ingredients observed during implementation of intervention and standard care, and (2) to describe the delivery and receipt of intervention and standard care components to understand how the intervention might have worked.

## Methods

### Participants

Two-hundred and eleven participants with type 2 diabetes were randomised to intervention or comparison groups in a ratio of two to one. Two-thirds (65.4%) were male, mean (standard deviation) (M (SD)) age was 63.2 (10.7) years, and the English Index of Multiple Deprivation score (0–100) was 10.3 (6.8). Patients were diagnosed with diabetes on average 6.8 (5.0) years ago and took a mean of 5.8 (2.5) different types of medication per day. HbA_1c_ (%) was 8.33 (1.24) and patients reported high adherence (M (SD) = 23.6 (2.5); potential range 5–25) [16]. Fifteen female practice nurses from 13 general practices delivered the consultations, of which 11 were lead nurse in diabetes care in their practice. The number of SAMS participants per practice ranged from 5 to 25 (median (inter-quartile range) = 8 (4 to 12)).

### Procedure

SAMS participants were recruited from 13 practices in Oxfordshire, Milton Keynes, Suffolk, Essex and Cambridgeshire (England, UK) following ethics approval (06/MRE02/3). Patients were eligible if they were aged at least 18 years, diagnosed with type 2 diabetes for at least three months, able to give informed consent, took any oral glucose-lowering medication, had HbA1c ≥ 7.5%, deemed by their general practitioner to be appropriate for tight glycaemic control, and were independent in medication taking. Consent was taken at a recruitment visit. Prior to the consultation with their usual practice nurse, patients were randomised centrally to the intervention (*n* = 126) or comparison group (*n* = 85). Nurses and patients consented to tape-recording the consultation and this was recorded on the tape. 194/211 consultations were tape recorded, 117 intervention and 77 comparison consultations. Ten patients did not attend and seven consultations were not tape-recorded. The introduction to the intervention was not recorded for two patients and the motivational component for one patient. Patients with and without tape-recorded sessions did not differ in gender (χ^2^(1) = 0.220; p = 0.639), age (t (209) = 0.665; p = 0.507) and self-reported medication adherence (t (199) = −0.045; p = 0.964) at baseline.

### *A priori* specified intervention and comparison consultations

Comparison patients attended a single 20-minute standard care consultation in which nurses took a blood sample for HbA1_c_ assessment (an indicator of diabetes control) and enquired about medications. Intervention patients received standard care plus the SAMS intervention in a single 50-minute consultation. Nurses introduced the intervention by mentioning that many patients do not take their medication as prescribed, and encouraging a non-judgemental discussion about medication taking. In the motivational component, nurses were expected to elicit patient beliefs about taking medication as prescribed based on the TPB [20], by asking questions about perceived benefits and disadvantages (instrumental and affective), (lack of) support, facilitators and barriers. They were expected to reinforce any positive beliefs by verbal and non-verbal acknowledgement and further exploration; and to prompt patients to problem solve any negative beliefs. In the action planning component nurses asked about current medication taking routines, explained that action plans might improve medication taking routines, and prompted patients to formulate and write down an ‘if… then…’ plan, also called an implementation intention [21], of where and when they would take each diabetes medication dose. Nurses were expected to read out any written plans aloud.

### Nurse training and quality assurance during the trial

A clinical psychologist and an intervention facilitator with a background in practice nursing (SB) delivered one-day training in the delivery of standard care and intervention components, supported by a manual and scripted protocols. Nurses were trained in the use of both motivational and action planning techniques. Intervention training also included patient-centred communication skills, *e.g.*, body language and active listening. The protocol did not prompt the use of these skills except open questions. Training methods were interactive and included practising the techniques, followed by feedback. To ensure consistent delivery across nurses and within nurses over time, research team members assessed audiotapes of all intervention consultations and purposively sampled comparison consultations during the entire period of intervention delivery. Standardised checklists were used to assess adherence to the scripted protocol, followed by oral and/or written feedback to the nurses.

### Assessment of intervention and comparison consultations

#### Development of the coding frame

We developed a single coding frame with detailed guidelines for intervention and comparison consultations. In the first phase, three researchers (WH, IK, ALK) identified variables informed by *a priori* hypothesised active ingredients and the content of the scripted protocols. Selection of variables was also informed by how nurses used the techniques in six tape-recorded consultations. The coding frame was developed and piloted in an iterative way (WH, IK, ADS). In the second phase, two researchers (LL, JG), one of whom (LL) had no prior knowledge of SAMS, listened to 11 tape-recorded consultations, using components from a grounded theory approach [22] to identify factors arising from the recorded data that might explain the effect of the intervention on medication adherence. The researchers listened to the recordings independently, identifying concepts which they then analysed thematically, comparing interpretations to produce a summary of the things that seemed important. The findings were discussed by the wider research team and following this, new variables reliably identified by listening to tape recordings were added to the coding frame which was piloted iteratively. Overlapping, unreliable and less relevant items were removed, with further adaptations (WH, LL, IK, ADS) and discussion at team meetings (all authors) until inter-rater agreement was at least 70%. Thirteen consultations were used to develop and pilot the coding frame. These were selected purposively to include intervention and comparison consultations, different practice nurses, and consultations of sufficient duration to be able to code as many elements as possible to test inter-rater agreement.

#### Assessment phase and inter-rater reliability

The main coder, a research psychologist (LL), assessed 117 intervention and 77 comparison consultations. Comparison consultations were coded in a single round and intervention consultations in two rounds: general ratings (*e.g.*, nurse adherence and competence) followed by specific ratings (*e.g.*, whether the nurse asked patients about perceived benefits of taking medication). For a robust reliability assessment, a second and third coder (IK, WH) double coded 49 (25%) randomly selected consultations between them, randomly allocated to each coder to balance any confounders (*e.g.*, trial arm). A further eight consultations were double coded (WH) along the duration of assessment to check for drift in the main coder. Any discrepancies were resolved and documented.

### Data analysis

Data were entered and analysed in PASW Statistics (18.0 and 21.0) for Windows. Double entry of a random sample of 20 consultations showed an error rate below 1%. Data checks were conducted for missing values and outliers. We used proportions for inter-rater agreement; due to restricted range of scores kappa values were low despite almost perfect agreement. Agreement was based on both presence and absence of variables. We used percentage general agreement for items assessed on a five-point Likert-type scale: instances of perfect or almost perfect agreement (one-point difference on a five-point scale) divided by the total number of observations. Percentage absolute agreement was used for all other items. Independent sample t-tests were used to investigate differences in nurse delivery between intervention and comparison groups. For the intervention group we created a total score for nurse delivery by summing the scores for standard care and intervention delivery and dividing by two.

## Results

### Phase one of coding frame development: *a priori* hypothesised active ingredients

We identified the following observable features of nurse delivery and patient receipt for inclusion in the coding frame (see Table 1).

**Table 1:** **Coding frame items informed by *a priori *** **hypothesised active ingredients and grounded theory**

**Items informed by a priori hypothesised active ingredients**		
**Nurse delivery**	**Number of items and scales**	**Inter-rater agreement**^**a,b**^
Protocol adherence	One item (5-point Likert type scale, ‘very poor’ (1) to ‘very good’ (5)).	93.9%
Competence	One item (‘very poor’ (1) to ‘very good’ (5)).	85.7%
Motivational techniques	Elicitation of patient beliefs: eight binary items (yes, no): (dis)advantages, good and bad feelings, (lack of) support, facilitators and barriers regarding taking medication as prescribed.	Elicitation: M = 99.5%; range 98.0-100.0%.
	Reinforcement and problem solving of relevant beliefs: eight items each (yes, no, not applicable if no beliefs to reinforce or problem solve).	Reinforcement: M = 78.6%; range 69.4-89.8%. Problem solving: M = 76.0%; range 69.4-87.8%.
Action planning techniques	Prompt elaboration of action plan for each dose taken: one item (‘very rarely/never’ (1) to ‘very frequently’ (5)).	M = 82.3%, range 77.6-87.8%.
**Patient receipt**	**Number of items and scales**	**Inter-rater agreement**^**a,b**^
Generation of beliefs	Eight binary items (yes/no). Items were scored as ‘yes’ if patients mentioned a relevant belief or said that they could not think of anything.	M = 92.6%, range 85.7-98.0%.
Formulation of action plans	Level of difficulty of each plan: one item (‘very difficult’ (1) to ‘very easy’ (5)).	M = 89.8%, range 87.8-93.9%.
	Vocal formulation of each plan: one item (‘to a very small extent’ (1) to ‘to a very great extent’ (5)).	M = 87.8%; range 85.7-91.8%.
	Who wrote each plan: one item (nurse, patient, unsure, other).	M = 94.6%, range 91.8-98.0%.
	Who read out each plan: one item (nurse, patient, unsure, other).	M = 93.2%, range 89.8-98.0%
	Read out in ‘if… then…’ format: one item (yes, no, not read out)	M = 93.9%, range 91.8-98.0%
	Number of changes in each plan compared to existing routines: one item (‘very few/none’ (1) to ‘very many’ (5))’	M = 96.6%, range 95.9-98.0%
**Items identified through grounded theory**		
**Nurse delivery**	**Number of items and scales**	**Inter-rater agreement**^**a,b**^
Communication style	Four items: professional/authoritative, friendly/relaxed, anxious/tentative, and angry/irritated (‘very rarely/never’ (1) to ‘very frequently’ (5)).	Intervention: M = 83.7%, range 71.4-98.0%
		Standard care: M = 73.5%, range 67.4-79.6%.
Positive and negative aspects of communication	Nine items: agreement, disagreement, rapport facilitation and inhibition, partnership facilitation and inhibition, giving information, counselling/directing behaviour, and social behaviour (‘very rarely/never’ (1) to ‘very frequently’ (5)).	Intervention: M = 83.5%, range 75.5-95.9%.
		Standard care: M = 69.6%, 55.1-85.7%.
Engagement with the intervention	One item (‘very disengaged’ (1) to ‘very engaged’ (5)).	93.9%
**Nurse-patient relationship**	One item for relationship quality (‘very poor’ (1) to ‘very good’ (5)).	Intervention: 91.8%
		Standard care: 73.5%
**Patient receipt of intervention**	Four items: engagement (‘very disengaged’ (1) to ‘very engaged’ (5)); amount of talk (‘very little’ (1) to ‘a lot’ (5)); question asking (‘very rarely/never’ (1) to ‘very frequently’ (5)); understanding (‘very poor’ (1) to ‘very good’ (5)).	M = 89.8%, range 83.7-93.9%.
**General items**	**Number of items and scales**	**Inter-rater agreement**^a, c^
**Duration**	Five items: standard care (two items), intervention introduction, motivational component, action planning component (minutes:seconds).	M = 92.0%, range 84.0-100%

Notes: ^a^Inter-rater agreement is reported for both coder pairs combined. It is expressed as percentage general agreement for items with Likert-type scales; and percentage absolute agreement for other items. ^b^Intervention: reliability during intervention delivery (*N* = 117). Standard care: reliability during standard care delivery to all patients (*N* = 194). ^c^Agreement between raters within one minute.

#### Nurse delivery of overall intervention and techniques

Two items were used for protocol adherence and competence of delivery. Competence was operationalised as encompassing faithful delivery of motivational and action planning techniques, fluency of delivery, use of patient-centred communication skills, and encouragement of a non-judgemental discussion. Contamination (not shown in Table 1) was assessed during the comparison consultations by coding any items in the coding frame related to intervention delivery, *e.g.*, motivational techniques. For the motivational component, we used eight items to assess whether nurses asked questions to elicit patients’ beliefs, which were summed to create an index of belief elicitation. We assessed whether nurses reinforced any positive beliefs and prompted problem solving of any negative beliefs; and calculated the proportion of patients for whom nurses reinforced beliefs or prompted problem solving. For the action planning component, we assessed to what extent nurses prompted patients to elaborate on their plan for taking each medication dose, whether nurses read out the action plans and whether they read them out in the specified ‘if…then…’ format.

#### Patient receipt of motivational and action planning techniques

For the motivational component, we assessed whether patients mentioned any beliefs relevant to taking medication, *e.g.*, better diabetes control as a benefit. We calculated the proportion of patients who mentioned relevant beliefs in response to each question. For the action planning component, we assessed how much difficulty patients experienced in formulating action plans, to what extent they vocalised them, whether they wrote down any action plans, and how much the action plans differed from their current routines. These were assessed for each morning, afternoon, or evening dose.

### Phase two of coding frame development: grounded theory approach

Two researchers (JG, LL) identified further aspects of nurse communication, nurse-patient relationship, and patient receipt of the intervention as potential active ingredients over and above those identified in phase 1. They were then operationalised in coding frame items.

#### Nurse communication

Provided that they reflected SAMS intervention content, we used items from reliable and valid instruments (Roter Interaction Analysis System (RIAS) [23], clusters of RIAS items [24]) and empirical studies [25,26]. We used four items to assess communication style, treated as individual variables as in previous studies [24-26]. Positive and negative aspects of nurse communication were assessed with nine items, treated as individual variables as they were not assumed to be opposite ends of a continuum. One item assessed nurse engagement with the intervention. All items were scored separately for delivery of the intervention and standard care.

#### Nurse-patient relationship

One item was used, scored separately for delivery of the intervention and standard care.

#### Patient receipt of the intervention

Four items assessed engagement, amount of talk, the extent to which patients asked questions and their understanding of intervention principles.

Finally, we coded the duration of standard care (the duration of the comparison consultation) and intervention delivery (introduction, motivational component and action planning component).

### Reliability of the coding frame during the assessment phase

The main coder did not show drift; inter-rater agreement was > 75% throughout the assessment period. For the robust reliability assessment, the scores of rater pairs (LL/IK and (LL/WH) were combined as overall agreement was > 75% for both pairs. Items on which both pairs showed < 75% agreement or differed more than 20% are highlighted below.

#### Reliability of phase one items

Mean inter-rater agreement was good (> 75%) for nurse protocol adherence, competence, delivery of motivational and action planning techniques, patients mentioning relevant beliefs about taking medication, and items assessing the formulation of action plans (see Table 1). Both pairs showed < 75% agreement on whether the nurse reinforced any beliefs about feeling good when taking medication as prescribed. Pairs differed more than 20% on how much patients vocalised plans for any evening doses.

#### Reliability of phase two items

When assessing nurse intervention delivery, mean inter-rater agreement was good (> 75%) for nurse communication, nurse engagement, quality of nurse-patient relationship, and patient receipt of the intervention. Pairs differed more than 20%, with one pair consistently showing lower reliability, on whether nurses inhibited rapport, gave information, counselled or directed behaviour, displayed social behaviour, and whether patients asked questions. When assessing delivery of standard care, inter-rater agreement on nurse communication was moderate to good. Both pairs showed < 75% agreement on whether nurses were professional/authoritative, showed agreement, facilitated rapport and partnership, counselled or directed behaviour, and displayed social behaviour. Pairs differed more than 20%, with the same pair showing lower reliability, on whether nurses were friendly/relaxed, anxious/tentative, showed agreement, facilitated rapport and partnership, and gave information.

### Nurse delivery of the intervention

#### Overall ratings

Nurses’ adherence to the scripted protocol was rated as good and competence in intervention delivery as neither poor nor good (see Table 2). On average, nurses showed a professional/authoritative and friendly/relaxed communication style. They frequently showed agreement, and occasionally facilitated rapport and partnership. Negative communication aspects were virtually absent. Over and above content of the protocol, nurses very rarely provided information and counselled or directed behaviour (*e.g.*, gave information about diabetes or dietary advice). Nurses and patients very rarely talked about issues unrelated to protocol content (social behaviour). Nurses’ engagement and the quality of their relationship with patients were rated as good.

**Table 2:** **Nurse delivery of the SAMS intervention (*n*** **= 117)**

**Overall ratings**	**M (SD)**^**a**^	**N**^**b**^
Protocol adherence	3.95 (0.91)	117
Competence	3.15 (1.01)	117
*Communication style*		
-Professional/authoritative	3.66 (1.15)	117
-Friendly/relaxed	3.14 (1.27)	117
-Anxious/tentative	1.28 (0.59)	117
-Angry/irritated	1.26 (0.54)	117
*Positive and negative aspects of communication*		
-Agreement	3.60 (1.17)	117
-Disagreement	1.12 (0.40)	117
-Rapport facilitation	2.92 (0.94)	117
-Rapport inhibition	1.16 (0.45)	117
-Partnership facilitation	2.93 (0.93)	117
-Partnership inhibition	1.44 (0.71)	117
-Giving information	1.50 (0.80)	117
-Counselling/directing behaviour	1.33 (0.60)	117
-Social behaviour	1.25 (0.62)	117
Engagement	3.57 (1.05)	117
Quality of relationship	3.71 (0.85)	117
**Delivery of behaviour change techniques**		
*Motivational techniques*		
Number of questions asked to elicit patient beliefs (0–8)	7.72 (0.62)	116
Proportion of patients for whom nurses reinforced positive beliefs^c^		
-Advantages of taking medication	64.7%	102
-Good feelings about taking medication	69.7%	66
-Others supportive of taking medication	77.2%	111
-Facilitators of taking medication	71.4%	70
Proportion of patients for whom nurses prompted problem solving of negative beliefs^c^		
-Disadvantages of taking medication	37.5%	40
-Bad feelings about taking medication	23.8%	80
-Others unsupportive of taking medication	25.0%	4
-Barriers to taking medication	21.2%	70
*Action planning techniques*		
Prompting patients to elaborate on action plans		
-Morning dose	2.50 (1.10)	103
-Afternoon dose	1.80 (0.96)	35
-Evening dose	1.96 (0.96)	98
Who read out the plan		
-Morning: 5.8% patient, 56.3% nurse, 37.9% not read out		103
-Afternoon: 11.4% patient, 51.4% nurse, 37.1% not read out		35
-Evening: 7.1% patient, 51.0% nurse, 41.8% not read out		98
Was the plan read out in the ‘if…then…’ format		
-Morning: 12.6% yes, 49.5% no, 37.9% not read out		103
-Afternoon: 2.9% yes, 60.0% no, 37.1% not read out		35
-Evening: 5.1% yes, 52.0% no, 42.9% not read out		98
**Duration of intervention (minutes: seconds)**		
Introduction	06:23 (02:20)	115
Motivational component	06:31 (03:08)	116
Action planning component	10:06 (04:00)	117
Total duration	23:00 (07:38)	115

Notes: ^a^Figures are means (standard deviations) and range is 1 to 5 unless specified. ^b^Number of participants with valid data. ^c^The denominator consists of all patients who were asked the specific question and mentioned a belief that could be reinforced or problem-solved.

#### Delivery of behaviour change techniques

During the motivational component, on average nurses asked almost all eight questions to elicit patient beliefs. The proportion of patients for whom nurses reinforced positive beliefs ranged from 64.7% of patients who mentioned advantages to 77.2% who mentioned that others supported them in taking medication. Nurses rarely prompted problem solving when patients mentioned negative beliefs or barriers, *e.g.*, changes in routines during weekends. The proportion of patients for whom nurses prompted problem solving ranged from 21.2% of patients who mentioned barriers to 37.5% who mentioned disadvantages. During the action-planning component, nurses occasionally prompted patients to elaborate further on plans for taking the morning dose, but rarely did so for any afternoon and evening doses. Around one-third of the plans were not read out by nurses, and of those that were, hardly any were in the specified ‘if…then…’ format. Nurses delivered no intervention components to the comparison group other than to one patient.

#### Duration

On average, the introduction and motivational component lasted just over six minutes each, and the action-planning component ten minutes. Mean total duration was 23 minutes, shorter than the specified 30 minutes [15].

### Patient receipt of the intervention

#### Overall ratings

Patients’ engagement and understanding of intervention principles were rated as good (see Table 3). They talked neither very little or a lot, and rarely asked questions.

**Table 3:** **Patient receipt of the SAMS intervention (*n*** **= 117)**

**Overall ratings**	**M (SD)**^**a**^	***N***^**b**^
Engagement	3.56 (1.03)	117
Amount of talk	3.29 (1.01)	117
Extent to which patients asked questions	1.35 (0.69)	117
Understanding of the intervention	3.48 (0.89)	117
**Receipt of behaviour change techniques**		
*Motivational techniques*		
Proportion of patients who mentioned relevant beliefs^c^		
-Advantages of taking medication	88.7%	115
-Disadvantages of taking medication	94.6%	112
-Good feelings about taking medication	80.7%	114
-Bad feelings about taking medication	90.0%	110
-Others supportive of taking medication	95.7%	116
-Others unsupportive of taking medication	98.1%	107
-Facilitators of taking medication	91.8%	110
-Barriers of taking medication	94.5%	110
*Action planning techniques*		
Perceived difficulty of generating action plans		
-Morning dose	3.77 (1.09)	103
-Afternoon dose	3.82 (1.06)	34
-Evening dose	3.94 (1.05)	97
Vocal formulation of action plans		
-Morning dose	4.22 (0.93)	103
-Afternoon dose	4.29 (0.72)	34
-Evening dose	4.09 (1.05)	97
Number of changes in plans compared to current routines		
-Morning dose	1.18 (0.62)	103
-Afternoon dose	1.06 (0.34)	
-Evening dose	1.28 (0.72)	97
Who wrote the plan down		
-Morning: 74.8% patient, 22.3% nurse, 2.9% unsure/other		103
-Afternoon: 68.6% patient, 28.6% nurse, 2.9% other		35
-Evening: 72.4% patient, 23.5% nurse, 4.0% unsure/other		98

*Notes*: ^a^Figures are expressed as means (standard deviations) and range of scores is 1 to 5 unless specified. ^b^Number of participants with valid data. ^c^The denominator includes all patients who were asked the question.

#### Receipt of behaviour change techniques

In response to the nurses’ questions about what patients thought about medication taking, the majority either mentioned relevant beliefs (*e.g.*, change in routines as a barrier) or said that they could not think of any benefits or barriers. This ranged from 88.7% of patients who mentioned something that made them feel good about taking medication to 98.1% who mentioned that others supported them in taking medication. Seventy-five (65.2%) intervention patients took two oral diabetes medication doses, 28 (23.9%) three doses, and 11 (9.6%) one dose. Only one patient (0.9%) took four doses including a second evening dose. One hundred and three patients made a plan for the morning dose, 35 for the afternoon dose and 98 for the evening dose. Patients seemed to find it easy to formulate plans for each dose, and vocalised them to a great extent. Across the three doses, an average of three-quarters of patients wrote down the plans. The plans differed very little from, or were identical to patients’ current medication taking routines.

### Differences in nurse delivery between intervention and comparison groups

Compared to the comparison group, nurses were more anxious and tentative in the intervention group, facilitated rapport and partnership less frequently, gave less information, counselled or directed behaviour less frequently and displayed less social behaviour (Table 4). Although the differences were marginally nonsignificant, there was a tendency for nurses to be less friendly and relaxed with the intervention group, inhibit partnership more frequently and have worse relationship quality with intervention patients. Nurses took less time to deliver the standard care component in the intervention (M (SD) = 11:42 (07:43)) than comparison group (M (SD) = 13:50 (06:06); t (187) = 2.031; p = 0.044).

**Table 4:** **Differences in nurse communication and relationship across the whole consultation between intervention and comparison patients (*N*** **= 194)**

	**Intervention *n*** **= 115**^**a**^	**Comparison *n*** **= 77**	**Mean difference between intervention and comparison (95% CI)**
	**M (SD)**	**M (SD)**	
*Communication style*			
Professional/authoritative	3.61 (1.06)	3.57 (0.85)	−0.042 (−0.327 to 0.244); t(190) = −0.288, p = 0.774
Friendly/relaxed	3.13 (1.18)	3.44 (1.03)	0.316 (−0.012 to 0.643); t(190) = 1.903, p = 0.059
Anxious/tentative	1.19 (0.44)	1.03 (0.16)	−0.161 (−0.265 to −0.058); t(190) = −3.065, p = 0.002
Angry/irritated	1.19 (0.44)	1.14 (0.45)	−0.044 (−0.173 to 0.085); t(190) = −0.673, p = 0.502
*Communication characteristics*			
Agreement	2.95 (0.97)	3.07 (1.18)	0.113 (−0.196 to 0.422); t(190) = 0.720, p = 0.472
Disagreement	1.10 (0.25)	1.07 (0.25)	−0.039 (−0.112 to 0.033); t(190) = −1.068, p = 0.287
Rapport facilitation	2.78 (0.88)	3.34 (0.98)	0.559 (0.291 to 0.828); t(190) = 4.117, p < 0.0001
Rapport inhibition	1.17 (0.36)	1.14 (0.39)	−0.031 (−0.139 to 0.077); t(190) = −0.565, p = 0.573
Partnership facilitation	2.47 (0.81)	2.79 (1.10)	0.323 (0.049 to 0.596); t(190) = 2.328, p = 0.021
Partnership inhibition	1.24 (0.38)	1.13 (0.50)	−0.114 (−0.238 to 0.011); t(190) = −1.801, p = 0.073
Gives information	1.93 (0.79)	3.10 (1.02)	1.174( 0.915 to 1.432); t(190) = 8.951, p < 0.0001
Counsels/directs behaviour	1.76 (0.75)	2.79 (1.21)	1.036 (0.757 to 1.314); t(190) = 7.342, p < 0.0001
Nurse and patient social behaviour	1.67 (0.69)	2.33 (1.21)	0.655 (0.384 to 0.926); t(190) = 4.769, p < 0.0001
*Quality of relationship*	3.77 (0.71)	3.97 (0.78)	0.205 (−0.010 to 0.418), t(190) = 1.885, p = 0.061

*Note*: ^a^Number of patients with valid data. Possible range of scores is 1 to 5.

## Discussion

This study shows that it is feasible to develop a reliable coding frame to assess the implementation of behavioural interventions such as those used in the SAMS intervention and standard care groups. Fidelity of intervention delivery and receipt were good. The findings provide insight into how the intervention might have worked and highlight challenges when nurses deliver behaviour change interventions. These are discussed in turn below.

We succeeded in developing a reliable coding frame for in-depth assessment of implementation of the intervention and standard care in the full trial sample. The coding frame was informed by both *a priori* hypothesised ingredients and also how the intervention was actually delivered in clinical practice. We are not aware of other studies that have used this combined method for in-depth assessment, drawing on the traditions of both sociology and psychology. Using components of the grounded theory approach, we identified nurse communication, nurse-patient relationship, and patient responses to the intervention as potential active ingredients beyond psychological mechanisms hypothesised *a priori*. These were congruent with the contents of the intervention and training and may affect patient outcomes. Patient-centred communication skills are considered foundation competencies for facilitating behaviour change [27]. Immediate patient responses to interventions may influence treatment adherence, as adherence is unlikely if patients cannot recall or understand behaviour change techniques taught [2].

The development of reliable measures for practitioner communication, relationship, and participant responses was challenging. Existing tools were designed to assess medical consultations, *e.g.*, RIAS [9], or specific interventions such as motivational interviewing [28], and are difficult to generalise to highly specified interventions. It proved more challenging to reliably assess the delivery of standard care than the intervention, perhaps because it was brief. Use of the grounded theory approach led to a comprehensive assessment that provided a rich insight into how the intervention was actually implemented, and helped to understand how it might have increased medication adherence.

We showed that the intervention and standard care were delivered and received well. The nurses were judged to show good adherence to scripted intervention protocols and contamination was virtually absent. Quality assurance may have played an important role in producing high adherence and low contamination in this study. The literature suggests that 80% to 100% represents high protocol adherence and < 50% low adherence [14]. However, few studies obtain fidelity levels over 80% and positive outcomes have been obtained with levels around 60% [12]. We assessed adherence on a Likert-type scale, but our measure indicates high protocol adherence when using these thresholds. Nurse competence of intervention delivery was rated lower than protocol adherence, confirming that high protocol adherence is not sufficient for competent delivery [29].

On average, the nurses were judged to use good communication skills during intervention delivery. They only occasionally facilitated rapport and partnership, which is likely to be due to the scripted protocol. The tape-recordings revealed that nurses delivered standard care in their own style, but use of the scripted intervention protocol resulted in greater formality and less fluency. This may have affected patients’ responses and interaction. Indeed, nurses used less patient-centred communication with intervention than comparison patients. A key reason may be that each nurse saw few intervention patients. They thus had little opportunity to become familiar with the intervention, deliver it faithfully in their own style, and tailor it to individual patients and medication taking problems. Other studies among practice nurses identified similar challenges with the use of scripted protocols, patient-centred communication and supporting patient self-management. The use of checklists by practice nurses in consultations with type 2 diabetes patients tended to decrease the flow and effectiveness of the consultation, particularly when nurses were less experienced and skilled [30]. Macdonald *et al.* found that nurses lacked resources to support patient self-management beyond personal experience and common sense approaches, such as giving information [31]. Kennedy *et al.* found that providing patient-centred communication and supporting patient self-management was challenging because practices did not perceive this as a priority, and nurses faced competing demands of achieving financially incentivised targets [32]. Patients were engaged and showed understanding of the intervention’s principles, but they rarely asked questions. It remains challenging in efficacy trials of behavioural interventions to achieve an optimal balance between standardisation across patients and practitioners and flexibility in delivery.

During the motivational component, nurses successfully elicited patients’ beliefs about taking medication and reinforced any positive beliefs. Whilst more than 80% of patients mentioned relevant beliefs, this included a majority of patients who said that they could not think of anything. Very few patients mentioned concerns or barriers, although we recruited patients whose elevated HbA1_c_ levels indicated suboptimal adherence. For these patients with established diabetes, taking medication seems a habitual rather than a reflective behaviour. Alternatively, patients may not have had adherence problems linked to motivation, or felt that sharing problems could damage the relationship with their usual practice nurse. When patients mentioned negative beliefs (*e.g.*, difficulties during the weekends), nurses rarely prompted patients to problem-solve them. This may be due to insufficient training, insufficient emphasis in the scripted protocol, time pressures, nurses failing to spot opportunities for problem solving, and nurse perceptions that problems were irrelevant (*e.g.*, past problems), infrequent (*e.g.*, holidays) or could be addressed by action planning.

Nurses seemed to find it easy to deliver the action-planning component and patients seemed to experience no problems in making action plans for each dose. The action plans were (almost) identical to current medication taking routines. Perhaps patients judged that their current routines were good, or the intervention failed to identify problems with routines. Indeed the plans tended to relate to patients’ normal routines, but not cover situations when their routine was disrupted, such as if they were away from home. There were some protocol deviations without clear reasons. Patients were expected to write down the plan, but nurses did this for a quarter of patients. Whilst nurses were expected to read out the plans, a third of plans were not read out.

The results show how the intervention may have increased medication adherence and generate hypotheses for future research. The intervention increased medication adherence with high fidelity, and was effective despite less patient-centred communication than in the comparison group and infrequent prompting of problem solving. Thus, we can exclude adaptations, patient-centred communication and problem solving as mechanisms of effect. More time discussing medication taking may have increased medication adherence. We hypothesise that another mechanism of increased medication adherence may be the visualisation and/or verbalisation of current routines, combined with formulating these as an action plan (*i.e.*, ‘action planning’). Visualisation maps onto ‘mental rehearsal of successful performance’ in the Behaviour Change Technique Taxonomy v1 [33]. Many patients described their current routines as a chain of behaviours resulting in them taking their tablets. A minority of patients read out their action plan, which could have acted as a commitment to take medication as prescribed.

The results illustrate the challenges for nurses when they deliver even brief behaviour change interventions in clinical practice. Although communication skills in intervention consultations were judged to be good, nurses showed less patient-centred communication than in the comparison group. More on-the-job training and rehearsal might facilitate the delivery of behaviour change techniques in a patient-centred, natural communication style. Training also needs to focus on problem solving techniques.

Our participant characteristics indicate that they were typical of people seen in routine primary care with type 2 diabetes, and our nurses reflect the kinds of nurses who manage people with type 2 diabetes. Therefore, our findings are likely to be generalisable to routine primary care.

Our recommendations include the identification of effective intervention components, development of fidelity measures, intervention design and potential adaptations. First, researchers need to identify critical, effective components of interventions to support medication adherence. Mental rehearsal of successful performance of the behaviour, action planning and commitment deserve further evaluation. Our next step is to identify critical components of the SAMS intervention by examining which aspects of nurse delivery and patient responses were associated with change in cognitive, behavioural, and clinical outcomes. Fidelity assessments could then focus on critical components and be less resource intensive. Second, we recommend more research on the development of reliable measures of practitioner communication, relationship and participant responses to behaviour change interventions. In order to assess factors beyond those hypothesised that may affect outcomes, we recommend that researchers identify these through recording or observing intervention contacts. Third, intervention developers need to observe usual practice and design interventions which are compatible with routine practice and adaptable to patients and local settings [34]. Such interventions are more likely to be implemented faithfully and have impact in the long term [12]. In this regard, the motivational component of the SAMS intervention included many questions and resembled oral administration of a Theory of Planned Behaviour questionnaire at a cost to patient-centred communication. It could be improved by asking patients on benefits and concerns about, and barriers to taking medication only. Furthermore, given the recommended ‘if…then…’ format in the literature about implementation intentions and supporting evidence, patients could be encouraged to read out their plans in this format.

## Conclusions

It is feasible to reliably assess the implementation of behaviour change interventions in clinical practice and this can provide insight into how interventions achieve any effects. Results could not be explained by poor delivery of motivational and action-planning components, definition of new action plans, or by improved problem-solving or patient-centred communication. Possible mechanisms of increased medication adherence include more time discussing it and mental rehearsal of successful performance of current routines, combined with action planning. Delivery of a new behaviour change intervention may lead to less patient-centred communication and possible reduction in overall trial effects.

## Competing interests

The authors declare that they have no competing interests.

## Authors’ contributions

WH was principal investigator, designed the study, participated in developing and piloting the coding frame, collected and analysed data, and wrote the manuscript. LL participated in developing and piloting the coding frame, collected all the data, analysed data, and participated in data interpretation. IK participated in study design, developing and piloting the coding frame, double-coding of consultations for the robust reliability assessment, and participated in data interpretation. ADS participated in developing and piloting the coding frame, collected data, and participated in data interpretation. JG participated in developing the coding frame and data interpretation. SB participated in data collection and interpretation. SS participated in data interpretation. AF participated in study design and data interpretation, and is co-PI of the SAMS trial. ALK provided senior advice on all aspects of study design, conduct, analysis, interpretation and writing up, and is co-PI of the SAMS trial. All authors read and approved the final manuscript.
